# The Two Faces of Cow’s Milk and Allergy: Induction of Cow’s Milk Allergy vs. Prevention of Asthma

**DOI:** 10.3390/nu11081945

**Published:** 2019-08-19

**Authors:** R. J. Joost van Neerven, Huub F. J. Savelkoul

**Affiliations:** 1 Cell Biology and Immunology Group, Wageningen University & Research, 6708 WD, Wageningen, The Netherlands; 2 FrieslandCampina, 3818 LE, Amersfoort, The Netherlands; 3 Allergy Consortium Wageningen, Wageningen University & Research, 6708 WD, Wageningen, The Netherlands

**Keywords:** cow’s milk allergy, milk, hydrolysate, asthma, processing, tolerance

## Abstract

Cow’s milk has been consumed by humans for over 5000 years and contributed to a drastic change in lifestyle form nomadic to settled communities. As the composition of cow’s milk is relatively comparable to breast milk, it has for a very long time been used as an alternative to breastfeeding. Today, cow’s milk is typically introduced into the diet of infants around 6 months, except when breastfeeding is not an option. In that case, most often cow’s milk based infant formulas are given. Some children will develop cow’s milk allergy (CMA) during the first year of life. However, epidemiological evidence also suggests that consumption of unprocessed, “raw” cow’s milk is associated with a lowered prevalence of other allergies. This Special Issue of *Nutrients* on “Cow’s Milk and Allergy” (https://www.mdpi.com/journal/nutrients/special_issues/milk_allergy) is dedicated to these two different sides of cow’s milk and allergy, ranging from epidemiology of CMA, clinical presentation and sensitization patterns, treatment and prevention, effects of milk processing, and current management guidelines for CMA, but also the epidemiological evidence linking cow’s milk to lower asthma prevalence as well as the tolerance-inducing effect of raw cow’s milk in food allergy models. In this editorial, we discuss these issues by highlighting the contributions in this Special Issue.

## 1. Cow’s Milk Allergy

The first recorded food allergy to dairy was described by Hippocrates (460–375 BC) that mentioned how some people could eat cheese without problems, but that eating cheese in some people causes pain [1]. As described in (Mucosal Immunology), Besredka described already in 1909 the process we now know as oral tolerance induction by performing experiments in laboratory animals. He demonstrated that oral (or rectal) administration of cow’s milk could prevent the development of anaphylaxis [2].

Cow’s milk allergy in children is characterized by the fact that most children will overgrow the allergy and develop immunological tolerance to cow’s milk before three to four years of age. Proper diagnosis and allergy management in early childhood is provided in the Diagnosis and Rationale for Action against cow’s milk allergy (CMA) (DRACMA) guidelines [3]. Extensively hydrolyzed milk formulas and amino acid formulas are often used to treat CMA in infants [4]. In the Middle East, the use of partial hydrolysates as an intermediate, first step-down from the use of intact cow’s milk formulas before introducing extensively hydrolyzed or amino acid formulas was recently discussed and approved during a consensus meeting [5].

In addition to CMA treatment—which in essence is the avoidance of ingestion of intact cow’s milk allergens—there is an urgent need to accelerate immunological tolerance to cow’s milk in these children. Recent progress in the understanding of the immunological basis of tolerance induction can aid in this development of novel sustainable therapies [4,6]. 

Oral tolerance is an active process of local and systemic immune unresponsiveness by which the immune system does not respond to an orally administered antigen such as food. The mechanism of this tolerance induction remains poorly understood but comprises the induction of regulatory T cell (Tregs) and also the induction of anergy or deletion of responding T-cell subsets [7]. 

Since these early descriptions of cow’s milk allergy (CMA) and oral tolerance induction, the prevalence of CMA in infants and toddlers has increased to approximately 0.5%–3% at 1 year of age, both in Western countries as in the rest of the world [8].

However, the prevalence of CMA, as the most common food allergy in early childhood, varies considerably based on the definition (i.e., parent-reported CMA > doctor-diagnosed CMA > double blind placebo-controlled food challenge (DBPCFC)-confirmed CMA), so the real prevalence may actually be lower than 1%–2%. Some children and even adults remain allergic to cow’s milk and often show severe reactions even to traces of milk. 

In bovine milk, caseins are the most abundant protein group, while whey proteins are more abundant in human breastmilk. In particular, β-lactoglobulin and αS2-casein are present in bovine milk but absent in human breastmilk and are important allergens in cow’s milk or preparations thereof.

The majority of patients with IgE-mediated CMA are sensitized to more than one milk allergen, with a great variability in the specificity and intensity of the induced IgE responses. Molecular-based allergy diagnosis allows associating each patient with a specific immune reactivity profile and potentially to identify different CMA phenotypes. Thus, different and novel diagnostic tests are needed to characterize the sensitization patterns of patients with different clinical pictures of CMA. Such tests comprise multiplex assays using purified (recombinant) allergen molecules as allergen arrays [4] to detect all potentially clinically relevant allergens and functional basophil activation tests to correlate to the severity of the allergic symptoms [9]. 

It is widely accepted that the prevalence of allergic sensitization and food allergy have increased over the last few decades. Still, it is unclear whether this also holds true for the development of CMA [8]. Moreover, high-risk children with CMA are more likely to also have multiple food allergies and suffer also from asthma, atopic dermatitis, and allergic rhinitis. These factors, in addition to the resolution of the CMA in many children, complicate the determination of the real prevalence. The susceptibility to developing CMA (and its persistence) also seems to be linked to differences in microbiota composition [10,11] and may also involve epigenetic components, which can explain the observed increase of food allergy prevalence worldwide. The described shared genetic etiology between CMA and asthma can thereby explain the associated sensitivity for subsequent development of asthma [12]. 

In contrast to sheep and goat’s milk that are very similar to cow’s milk, milk derived from camels and cow’s milk have a low cross-reactivity, which is indicative of a low protein similarity. As such, camel milk may be an alternative to cow’s milk-based hypoallergenic infant formulas for children at high risk of developing CMA [13]. In addition to the most frequent type I or IgE-mediated allergy against cow’s milk, also non-IgE-mediated milk allergy exists. Often these patients show a delayed onset of disease after allergen exposure. The mechanism of this delayed-type hypersensitivity reaction is poorly understood, and a mouse model can help to improve our understanding [14]. 

## 2. Milk Processing and CMA

Until the industrial revolution and the founding of milk processing factories, milk was mostly consumed in its raw or boiled form. Since the 1880s, milk pasteurization and higher heat-treated milks have become the industrial standard to prevent the spread of milk-borne pathogens that can cause *a.o.* diarrhea. Various heat treatments, including pasteurization and ultra-high temperature, are widely used today to increase the safety of raw milk and extend its shelf life. 

Thermal treatments will also lead to structural changes of the milk proteins, including protein aggregation and glycation. These protein alterations can thus modulate the cow’s milk protein immunogenicity (the induction of specific IgE production) and/or allergenicity (the ability to cross-link cell-bound IgE on mast cells with specific allergens, provoking the release of mediators, including histamine) [15]. Such aggregated and glycated proteins can interact with multiple receptors on immune cells linking the allergic sensitization profile to individual clinical responsiveness when exposed to cow’s milk allergens [16].

Surprisingly, it was found that most children with CMA can tolerate baked milk, and such children appeared to have lower β-lactoglobulin, and casein-specific IgE concentrations and higher numbers of regulatory T (Treg) cells present in their circulation [6,17]. 

Baked milk (as within the muffin matrix) might also promote formation of complexes with food components, inducing a modulation of the immune reactivity and reduction of allergenicity of milk allergens. Addition of baked milk products into the daily diet can accelerate the induction of tolerance to unheated raw milk rather than complete avoidance of the allergenic food [18]. Regular ingestion of baked milk products could thus drive a change in immune responsiveness, thereby inducing milk tolerance. 

Allergy prevention was for a very long time based on allergy avoidance measures. 

Currently, however, actively interfering with influencing immune tolerance based on novel insights into the (heat treatment modified) hypoallergenic allergen molecules, the use of (partially) hydrolysed formula’s, the epitope-specificity of the IgE antibodies, the shifting balance between T-cell subsets, and the induction of Tregs are considered to be more effective in prevention of the development of allergic symptoms [6,17]. 

## 3. Raw Milk and Allergy: Evidence from Epidemiology and Animal Models

Interestingly, in the early 2000s, milk was associated with allergy in quite a different and unexpected way. Searching for an environmental link with asthma, children growing up on small farms were found to have a much lower risk for developing asthma and allergic rhinitis [19]. In follow-up studies, it was shown that this effect was independently linked to exposure to the farming environment (stables and animals) on the one hand and to consumption of unprocessed (raw) cow’s milk on the other hand [20,21,22]. To date, more than 15 epidemiological studies have shown that the consumption farm milk, most of which is consumed as raw unpasteurized cow’s milk, can reduce the risk of allergic diseases. This has been reviewed in Sozanska in this issue [23] and in [24]. 

These results have now been confirmed by causal evidence in mouse model systems [25,26]. In this issue, these authors demonstrated that the suppression of food allergic symptoms by raw cow’s milk is retained after skimming but abolished after pasteurization of the milk, and subsequent addition of alkaline phosphatase might restore the allergy-protective effects. In a follow-up paper, the same group addressed the role of epigenetic modification as part of the mechanism of action [27]. This is in line with the finding that exposure to raw farm milk in pregnancy and the first year of life induces epigenetic changes in innate immunity receptor genes [28]. 

As a further mechanism, the consumption of immunomodulatory cytokines in unprocessed bovine milk may create the environment to promote the development of regulatory T cells, enabling establishment and maintenance of oral tolerance in the gut, a process in which bovine IgG may also be involved through the formation of allergen–IgG complexes [29,30]. Such IgG–allergen-immune complexes in murine milk have been shown to protect against allergies in experimental models [31,32].

## 4. Conclusions

**Figure 1:**
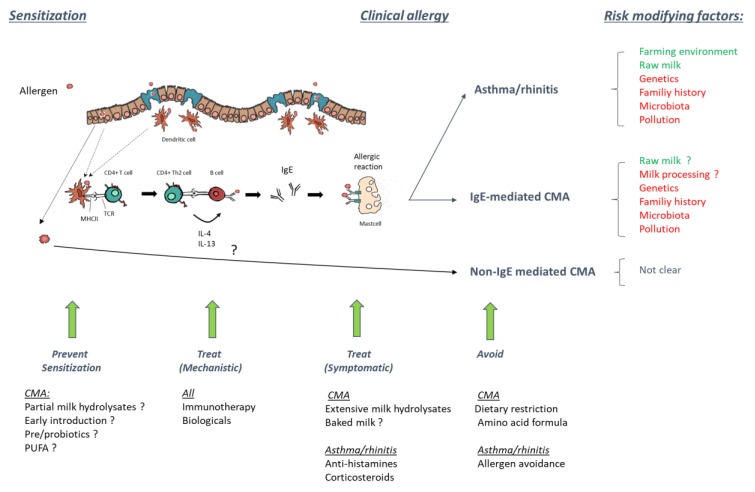
Sensitization, risk factors, and current strategies to prevent and treat allergies (cow’s milk allergy (CMA) as well as inhalation allergies).

We hope this Special Issue provides a state-of-the-art overview of the two-faced story of cow’s milk and allergy, on the one hand, by describing how early introduction, milk processing, milk protein hydrolysates, and new immune-therapeutic approaches can help to prevent and manage CMA in the clinic, and on the other hand, highlighting how consumption of raw or minimally processed milk might become relevant in future preventive strategies for respiratory and possibly food allergies (see Figure 1 for overview).
